# A longitudinal resource for mapping interindividual variation in the aging connectome

**DOI:** 10.1038/s41597-026-07746-7

**Published:** 2026-07-30

**Authors:** Anna MacKay-Brandt, Yunglin Gazes, Daniel Garcia-Barnett, Lauren A. Grebe, Olivia Ripley, Kai Xuan Gan, Kristin D. Trautman, Melissa Kramer, Melissa M. Breland, Russell Tobe, Alexandre R. Franco, Vilma Gabbay, Michael Milham, Stan J. Colcombe

**Affiliations:** 1 Brain Aging and Mental Health Laboratory, Clinical Research, Nathan Kline Institute, Orangeburg, NY USA; 2 Center for Biomedical Imaging and Neuromodulation, Nathan Kline Institute, Orangeburg, NY USA; 3 Communication Sciences and Disorders, St. John’s University, Queens, NY USA; 4 Clinical Research, Nathan Kline Institute, Orangeburg, NY 10962 USA; 5 Child Mind Institute, New York, NY USA; 6 Department of Psychiatry, New York University Grossman School of Medicine, New York, NY USA; 7 University of Miami Leonard M. Miller School of Medicine, Miami, FL USA

## Abstract

Trajectories of age-related neurocognitive decline are nonuniform, and are impacted by numerous environmental and physiological factors. Earlier life phases set the stage for later life neurocognitive function, with midlife marking a critical transition characterized by increasing variability in cognitive, affective, and physiological functioning. Despite its importance, this turbulent period remains underrepresented in open neuroimaging data resources. To address this gap, the Nathan Kline Institute - Rockland Sample (NKI-RS) created ‘Mapping Interindividual Variation in the Aging Connectome’ (MIVAC), an openly shared, multimodal dataset designed to map brain aging trajectories beginning in midlife and assess the influence of key modifiable factors linked to dementia prevention such as cardiorespiratory fitness, sleep, and mood. This longitudinal investigation includes 348 community-ascertained participants aged 38 to 71 years at baseline, with 219 participants completing 3 annual timepoints. Data collection incorporated deep phenotyping, including detailed assessment of cognitive, behavioral, medical, and cardiorespiratory fitness domains, to compliment multimodal neuroimaging (resting-state fMRI, diffusion MRI, morphometric MRI, and arterial spin labeling) and biospecimen collection. The protocol harmonizes with prior NKI-RS sub studies, enabling lifespan cross-sectional or longitudinal questions, while incorporating age-specific considerations for cognitive and neural aging. The full dataset is openly available.

## Background

Psychiatric and cognitive disorders are prevalent throughout the lifespan and pose a significant burden on individuals, families, and public health systems. Considerable recent effort has been directed toward understanding disruptions in brain function underlying early-onset psychiatric illness^1–4^ and late-life cognitive decline^3^. Midlife, however, remains a relatively underrepresented period in lifespan neuroscience research^5^, despite the emerging recognition that it sets the stage for later life neurocognition^6–8^. This stage of life, typically defined as the period between the fourth and seventh decades, is characterized by increased variation both between and within individuals in cognitive^9,10^, brain^11^, and physiological^12^ functioning. It is also a critical period in which modifiable risk and protective factors may influence later life brain health and cognition^13,14^. Yet few open-access neuroimaging datasets exist that characterize brain structure, function, and behavior across mid-to-late-life, either cross-sectionally^15^ or longitudinally^16,17^. The Nathan Kline Institute - Rockland Sample (NKI-RS) - Mapping Interindividual Variation in the Aging Connectome (MIVAC) sub study was designed to emphasize cardiorespiratory fitness and physiological function across multiple timepoints to understand the impact of natural variation of this key modifiable factor on brain and cognitive function in community samples.

Cardiorespiratory fitness (CRF) is a well-established predictor of cognitive health in mid-to-late life. Meta-analytic evidence indicates that aerobic exercise disproportionately benefits executive function relative to other cognitive domains^18^, and higher CRF has been linked to greater brain volume in prefrontal and hippocampal regions^19,20^, preserved white matter microstructure^21^, and enhanced cerebral perfusion^22^. Together, these findings point to CRF as a modifiable factor that supports the neural architecture underlying cognitive aging. However, CRF does not operate in isolation; sleep quality, anxiety, and depression may independently and interactively influence executive function trajectories^23,24^, yet few datasets are designed to examine these relationships jointly. Existing mid-to-late-life cohorts that map cognitive change trajectories typically rely on brief screening instruments or limited cognitive batteries that treat executive function as a unitary construct. The present dataset addresses this gap by pairing CRF and mental health assessments with a detailed characterization of executive function enabling researchers to investigate how fitness and psychological well-being relate across or within specific executive subprocesses.

The current dataset was designed to complement the previously described pediatric longitudinal protocol of the NKI-RS^4^, which emphasized brain development and the emergence of psychiatric illness from ages 6 to 17. This sub study extends the community-ascertained, transdiagnostic, and deeply phenotyped framework of the NKI-RS program^25^ for a longitudinal characterization of interindividual variation across midlife and older adulthood (ages 38–71). The MIVAC sub study supports efforts to map normative and atypical brain aging trajectories and to evaluate modifiable determinants of cognitive and neural health. Specifically, this study incorporates cardiorespiratory fitness assessment, medical and behavioral phenotyping, and multimodal imaging to investigate risk and protective factors that affect brain health and cognition.

To achieve these goals, we implemented a multi-cohort longitudinal design, prospectively harmonized our core protocol with prior NKI-RS sub studies, and maintained the NKI-RS commitment to open science and data sharing. Imaging protocols include multiband functional and high-angular resolution diffusion magnetic resonance imaging (MRI), arterial spin labeling, and structural morphometry^4^. Comprehensive phenotyping includes psychiatric diagnostic interviews, cognitive and behavioral assessments, medical history, laboratory tests, physical measures, and the addition of gold-standard cardiorespiratory fitness assessment (VO_2_max). Protocol harmonization across prior lifespan cross-sectional and pediatric longitudinal NKI-RS sub studies allows for unique, rich characterizations across lifespan neuroimaging analyses. This data descriptor outlines the rationale, design, implementation, and available data for the NKI-RS MIVAC cohort and provides guidance for researchers interested in the use of these data for the study of brain aging from midlife to older adulthood.

## Methods

### Recruitment and Retention Strategy

The target enrollment was 434, with 400 participants recruited between January 2016 and April 2018. The last follow-up assessment completed before the coronavirus pandemic-related shutdown was in March 2020. Recruitment followed the strategies established across all NKI-RS sub studies to approximate a community-representative enrollment for the local region. Detailed recruitment descriptions common to all NKI-RS studies can be found in prior reports^4,25^.

In addition to program-wide approaches, the MIVAC sub study included unique recruitment, retention, and feedback strategies. Study investigators with expertise in healthy aging hosted community talks at NKI and in local community spaces. Limited clinically-relevant feedback was provided to participants upon request to support the individual health goals of our participants when shared with their healthcare providers (See^4^ for more detail). However, due to concerns about sampling bias, feedback was not highlighted as part of MIVAC study recruitment and potential participants who stated concern about memory impairment were referred for a free memory evaluation through the NKI Geriatric Psychiatry Research Program and not enrolled.

Building upon lessons learned from three preceding NKI-RS sub studies^4,25,26^, procedures adopted or implemented to promote retention included:

- Birthday cards sent to participants to help maintain a positive connection and track address changes.
- Open house events and “coffee talks” hosted by the study investigators and designed to create a dialogue to translate preliminary findings, report on study progress, and answer any general questions about healthy aging raised by the participant community.
- Newsletters emailed to participants.
- Written and verbal participant satisfaction feedback collected at each study visit.
- Prior to MIVAC data collection, the core protocol across all prior sub studies was reviewed by the NKI-RS leadership team and reduced to increase efficiency and decrease participant burden.

**Fig. 1:**
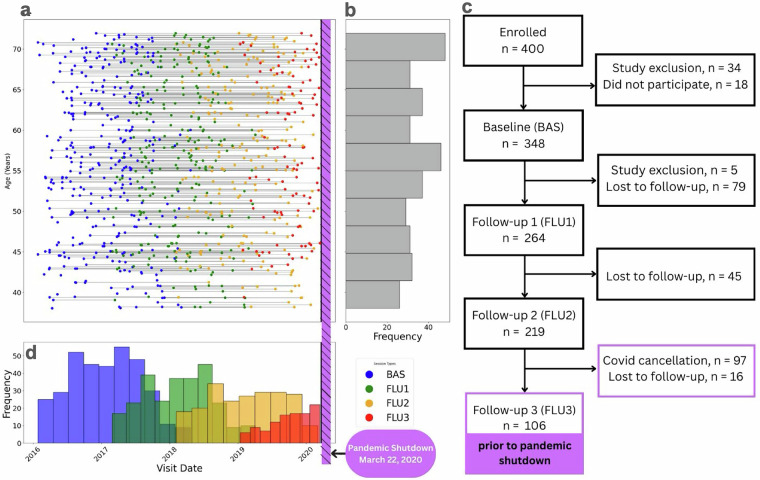
Overview of participant age distribution and retention. (**a**) Timeline for each participant’s visits plotted against their age. Each visit is color-coded for visit number. (**b**) Histogram of participant age. (**c**) STROBE reporting diagram for observational studies. (**d**) Histogram of each visit. Hatched purple bar indicates the date of pandemic shutdown on March 22, 2020. 285 out of 348 participants from BAS (82%) participated in the COVID survey during the pandemic shutdown.

Of the 400 participants enrolled, 348 (87%) completed a baseline visit. Among those who completed baseline visits, 5 (1.4%) were identified to have an MRI contraindication (e.g., presence of ferromagnetic implants, pacemakers, cerebral pathologies such as a lifetime history of stroke or traumatic brain injury, or a TIA in the past year, and other standard MRI safety exclusions). Of the remaining participants, 264 (77%) completed a first annual follow-up. A second annual follow-up was completed by 219 (64%) participants. The coronavirus pandemic interrupted the third annual follow-up period, with 106 (31%) completed, 16 (5%) due for assessment but lost to follow-up, and 97 (28%) were scheduled but administratively canceled for health safety reasons related to pandemic precautions. While this sub study reached its endpoint at the start of the pandemic, please note that 285 (82%) study participants completed an online survey extension study during the first three months of the coronavirus pandemic-related shutdown (May 2020 - August 2020) reporting on the impact of the public health mandated shutdown on their daily life activities, physical and psychological health. See Fig. 1 for visualizations of age by visit and a Strengthening the Reporting of Observational Studies in Epidemiology (STROBE) reporting diagram for observational studies^27^. There were no age, sex, race/ethnicity, level of education, or WASI full scale percentile rank differences between the baseline cohort and those participants who completed at least three longitudinal timepoints.

### Participant Procedures

All NKI-RS sub studies followed the same procedures, with staff trained across all contemporaneous studies and supervised by the NKI-RS leadership team. Common procedures are detailed elsewhere^4,25^ and available on the NKI-RS website (https://rocklandsample.org/for-researchers). The MIVAC sub study specific procedures are described in detail below.

#### Screening

**Fig. 2:**
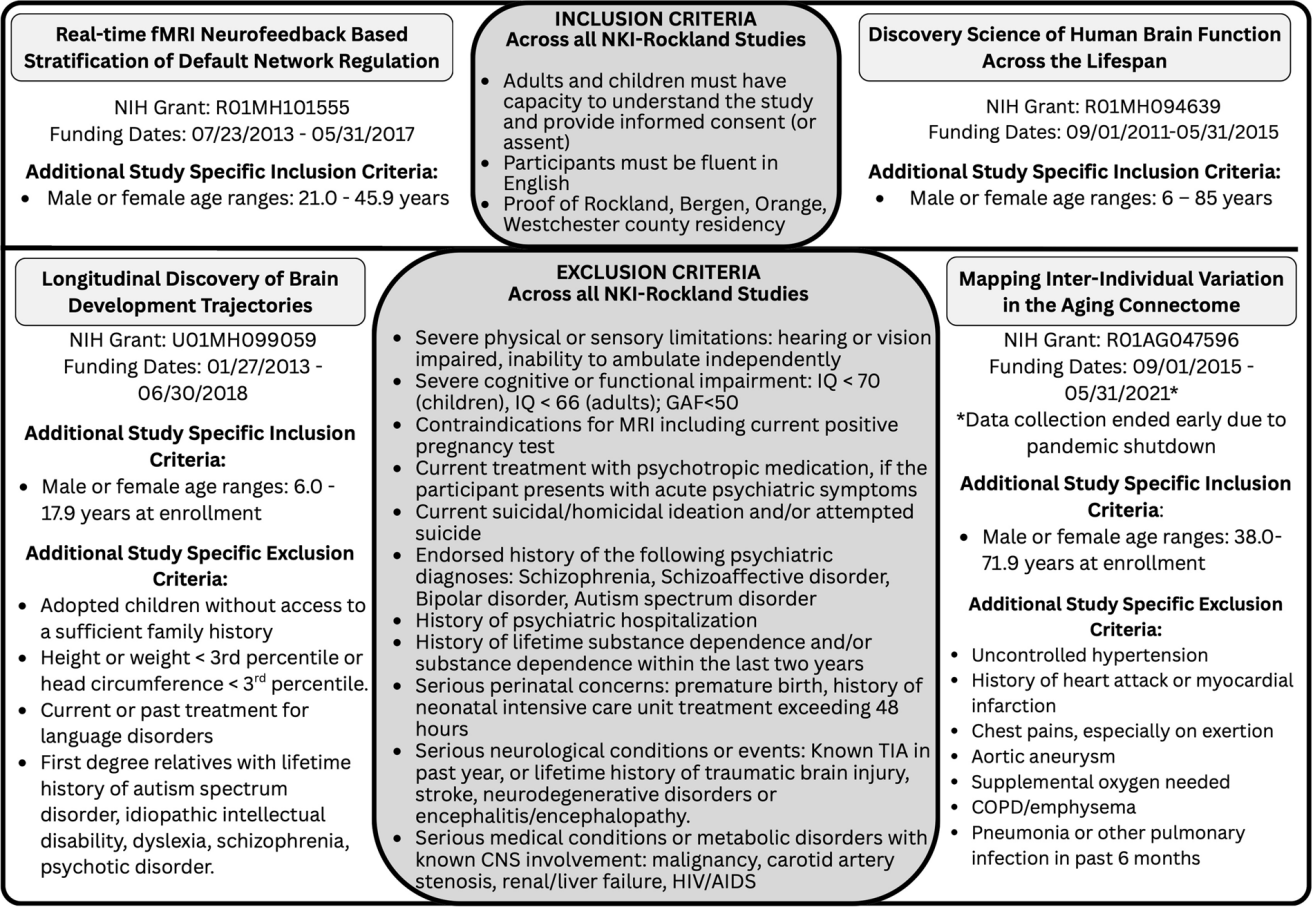
Inclusion and exclusion criteria for the aggregate NKI-RS cohort and each substudy. Shared selection criteria among the four studies are presented in the center boxes. Study specific criteria are listed under each NKI-RS sub study.

Potential participants completed a pre-screening phone interview with an intake coordinator. The screening interview assessed eligibility across all NKI-RS sub studies according to common and study specific inclusion criteria (See Fig. 2). Individuals meeting study criteria were invited to participate.

#### IRB approval

This study was approved by the Nathan Kline Institute for Psychiatric Research Institutional Review Board (IRB protocol number 790801). Prior to conducting the research, written informed consent was obtained from the participants for study participation and open sharing of de-identified data and biological specimens through archival data repositories (See Data Records section for details). Participants were informed that they could withdraw consent and discontinue participation at any time. Participants were informed that they may request to have data removed from data sharing. Any data released before this request would not apply. Participant information is stored on secure study servers and in locked cabinets at the Nathan Kline Institute. Personally identifiable information is removed from all shared datafiles and handled only by study staff. Random 5 digit identifiers were assigned to all data with linking information stored on secure study servers. All methods were carried out in accordance with relevant institutional, NY State, and federal guidelines and regulations.

#### Experimental Design

The MIVAC study design included 9 visits across four years. In the first year, participants attended a brief consent visit, a baseline full-day characterization visit, and a separate one-hour cardiorespiratory fitness assessment (median lag between baseline visit and fitness assessment was 8 days). Each annual follow-up included separate characterization and cardiorespiratory fitness assessment visits (median lag between follow-up visit and fitness assessment was 6 days). All study visits, within each timepoint, were conducted on consecutive days, when possible, with a goal of no more than 2 weeks separation from first to last visit. Cognitive testing and MRI were typically administered on the same day (median day lag = 0). Adjustments to the schedule were made to complete assessments on an additional visit when necessary to decrease participant burden. Datasets include a variable that codes for interval time between each assessment (i.e. “day lag”) to allow researchers greater precision when considering analyses within a data collection timepoint (i.e. baseline or follow-up epoch). Follow-up visits included nearly all the same assessments as the baseline visit with a few exceptions. See Table 1 for a list of all assessments, indicating if collected at baseline, follow-up, or both, and noting if the assessment is unique to the MIVAC sub study. All assessments without notation are also collected as part of the common NKI-RS aggregate lifespan dataset. An example schedule is provided in Table 2. Please note, MRI data collection was typically scheduled for late morning or early afternoon and remained relatively constant across study visits. Research staff attempted to adjust start and end times relatively earlier or later based on individualized feedback when scheduling to minimize time of day effects. See also https://rocklandsample.org/mivac-full-end-user-protocol-2 for full details.

**Table 1:** Assessments by visit.

Assessments	Base-line	Follow-Up	Assessments	Base-line	Follow-Up
**General**			**Imaging**		
Brain Injury Screening Questionnaire (BISQ)	✓	✓	MRI	✓	✓
Consent	✓	✓	MRI-Questionnaire	✓	✓
Demographics Questionnaire	✓	✓	**Diagnostic**		
Demographics Supplement	✓	✓	Adult ADHD Clinical Diagnostic Scale (ACDS)	✓	
Family History Questionnaire	✓		Global Outcome Scale (GOS; 65 + )	✓	✓
Hollingshead Socioeconomic Status (SES)	✓		Structured Clinical Interview for DSM Disorders (SCID)	✓	✓
Medical History Questionnaire	✓	✓	**Behavioral Tasks**		
Medications/Medical Conditions	✓	✓	Rapid Visual Information Processing (RVIP)	✓	✓
Satisfaction Survey	✓	✓	**Behavior and Phenotypic Characterization Questionnaires**		
Social Networking Questionnaire	✓	✓	Adult Self Report / Older Adult Self Report Adaptive Functioning (ASR-AF/OASR-AF)	✓	✓
Zipcodes	✓	✓	Adult Self Report / Older Adult Self Report (ASR/OASR)	✓	✓
**Physical**			Adult Temperament Questionnaire (ATQ)	✓	✓
6-Minute Bike Test	✓		Beck Depression Inventory (BDI - II)	✓	✓
Actigraphy	✓		Cognitive Failures Questionnaire (CFQ)	✓	✓
Blood Collection	✓	✓	Conners Adult ADHD Rating Scale – Self Report, Short Version (CAARS)	✓	✓
Cambridge-Hopkins Restless Legs Syndrome (CHRLS)	✓	✓	Domain-Specific Risk-Taking Scale (DOSPERT)	✓	✓
Color Vision	✓		Eating Disorder Examination Questionnaire (EDEQ)	✓	✓
Edinburgh Handedness Questionnaire (EHQ)	✓		Emotional Regulation Questionnaire (ERQ)		✓
Grip Strength	✓	✓	Inventory of Callous-Unemotional Traits (ICU)	✓	
Grooved Pegboard	✓	✓	Interpersonal Reactivity Index (IRI)	✓	✓
International Physical Activity Questionnaire (IPAQ)	✓	✓	NEO Five Factor Inventory (NEO-FFI-3)	✓	✓
Pittsburgh Sleep Quality Index (PSQI)	✓	✓	Physical Activity Scale for the Elderly (PASE)	✓	✓
VO2 Submax Testing*	✓	✓	21-Item Peters *et al*. Delusions Inventory (PDI-21)	✓	✓
Walking While Talking (WWT)*	✓	✓	PhenX Sexual History	✓	✓
Weight/Height, Vital Signs, Hip/Waist Measurement	✓	✓	Penn State Worry Questionnaire (PSWQ)		✓
**Cognitive**			Perseverative Thinking Questionnaire (PTQ)		✓
Attention Network Task (ANT)	✓	✓	Ruminative Response Scale (RRS)	✓	✓
Digit Span	✓	✓	Sex Role Identity Scale	✓	
Delis-Kaplan Executive Function System (DKEFS) - Color-Word	✓	✓	Sexual Orientation Scale	✓	
DKEFS - Design fluency	✓	✓	State Trait Anxiety Inventory (STAI)	✓	✓
DKEFS - TOWRE	✓	✓	Three-Factor Eating (TFEQ)	✓	✓
DKEFS - Trails	✓	✓	Trauma Symptom Checklist for Adults (TSC-40)	✓	✓
DKEFS - Verbal Fluency	✓	✓	University of California at Los Angeles Posttraumatic Stress Disorder Reaction Index (UCLA-PTSD)	✓	✓
Montreal Cognitive Assessment (MoCA)	✓	✓	UPPS-P Impulsive Behavior Scale (UPPS)	✓	
Penn Computerized Neurocognitive Battery (CNB)	✓	✓	**Substance Use and Addiction Measures**		
Rey Auditory Verbal Learning Test (RAVLT)	✓	✓	Comprehensive Addiction Severity Index for Adolescents (CASI-A)	✓	✓
Wechsler Abbreviated Scale of Intelligence (WASI-II)	✓	✓	Fagerstrom Test for Nicotine Dependence (FTND)	✓	✓
Wechsler Individual Achievement Test – Second Edition Abbreviated (WIAT-IIA)	✓		National Institute on Drug Abuse Questionnaire (NIDA)	✓	✓
**Assessments unique to MIVAC Study**			Urine Drug Test	✓	✓

**Table 2:** Sample Baseline Characterization Schedule.

Duration (mins)	Activities
First Visit - Consent, Brief Cognitive and Health Screening (1 hour)	
20	Consent
20	Cognitive Screening - MoCA
10	Health Screening - Morphometrics
10	Set up at-home surveys
Second Visit - Main Protocol (Full day)	
10	Phlebotomy
15	Breakfast
90	Experimental cognitive tasks
10	Break
90	Neuroimaging
30	Lunch
90	Neuropsychological assessment
60	Psychiatric diagnostic interview
30	Psychosocial/health surveys
15	6-min bike assessment
Third Visit - Fitness assessment (1 hour)	
15	Walking While Talking procedure
30	VO_2_ Submaximal Cardiorespiratory Fitness

Annual follow-up appointments maintained the same visit schedule, see Table 1 for follow-up assessment details.

#### Clinical Assessments

Detailed procedures for clinical assessments common across the NKI-RS sub studies can be found in^4^ and https://rocklandsample.org/mivac-full-end-user-protocol-2. Please note that some self-report assessments developed for specific age cohorts were extended to younger or older ages across the NKI-RS aggregate sample (e.g., Montreal Cognitive Assessment: MoCA, Comprehensive Addiction Severity Index for Adolescents: CASI-A), while age-specific versions of measures were also chosen and matched to the participant age (e.g., Achenbach System: Adult Self Report / Older Adult Self Report: ASR, OASR; Geriatric Depression Scale: GDS). The procedure was to assign sets of surveys to participants based on age that balanced breadth of coverage for the aggregate NKI-RS lifespan sample while also recognizing the importance of age-specific framing for some measures. Note that some items of the ASR and OASR overlap, allowing for item-level coverage across middle to older adulthood. See: https://rocklandsample.org/mapping-interindividual-variation-in-the-aging-connectome-mivac for item-level documentation.

Within the NKI-RS research program, the characterization of global cognitive function using the MoCA^28^ was unique to the MIVAC sub study. Research technicians, under the supervision of the study neuropsychologist, administered the MoCA at baseline and each annual follow-up visit. Administration and scoring followed the standardized protocol. All scores below 26 were sent to the neuropsychologist for review. Scores were compared with all other available study data and any contextual information. The study neuropsychologist noted if there was or was not any indication of dementia based on a full chart review. If there was an indication of possible dementia at baseline, the participant was flagged for exclusion. Otherwise, the participant was included.

#### MRI

All NKI-RS sub studies shared the same core neuroimaging protocol, and collection was performed using the same 3.0 T Siemens TIM Trio scanner at the Nathan Kline Institute with a 32-channel head coil for all acquisitions. During each imaging session, participants underwent a comprehensive protocol consisting of two morphometric sequences (T1-weighted MPRAGE and T2-SPACE), one diffusion MRI sequence, six functional MRI runs (three resting state with different acquisition parameters, one breathholding, two visual checkerboards, and one pseudo-continuous arterial spin labeling [pCASL]), and a T2-FLAIR sequence. The three resting-state runs were collected within the same session to enable methods comparison: a TR 1400 ms multiband sequence (2 × 2 × 2 mm) emphasizing spatial resolution, a TR 645 ms multiband sequence (3 × 3 × 3 mm) emphasizing temporal resolution, and a conventional CAP sequence (TR 2500 ms; 3 × 3 × 3 mm) serving as a well-established ground-truth reference for evaluating the multiband acquisitions (Note: This protocol was established as part of the core NKI-RS imaging protocol in 2011 when multiband sequences were not yet publicly available. We collected the range of fMRI scans to facilitate the objective evaluation of multiband imaging across the lifespan.). Imaging parameters are presented in Table 3. See also^4^ for a detailed description. Full imaging protocols can be downloaded here: https://rocklandsample.org/for-researchers-rockland-sample-i-2011-2022-neuroimaging-protocol

**Table 3:** Parameters for MR Sequences.

Scan Type	Time Acquisition (min:sec)	Slices	% FOV Phase	Resolution (mm)	matrix	TR (ms)	TE (ms)	Flip Angle (°)	Multi Band Accel	Phase Partial Fourier	Notes
T1-weighted (MPRAGE)	4:18	176	96%	1 × 1 × 1	256 × 256	1900	2.52	9	N/A	off	TI (ms) = 900
T2 FLAIR	3:02	44	75%	1 × 1 × 3	256 × 192	900	106	180	N/A	off	TI (ms) = 2500
dMRI	5:43	64	84.90%	2 × 2 × 2	106 × 90	2400	87	90	4	6/8	128 directions; b = 1500 s/mm2; 9 b0 images
R-fMRI (mb-645)	9:46	40	100%	3 × 3 × 3	74 × 74	645	30	60	4	off	
R-fMRI (mb-1400)	9:45	64	100%	2 × 2 × 2	112 × 122	1400	30	65	4	6/8	
R-fmri (sb)	5:05	38	100%	3 × 3 × 3	72 × 72	2500	30	80	sb	off	
Visual Checkerboard Stimulation (CB-645)	2:41	40	100%	3 × 3 × 3	74 × 74	645	30	60	4	off	
Visual Checkerboard Stimulation (CB-1400)	2:27	64	100%	2 × 2 × 2	112 × 112	1400	30	64	4	6/8	
Breath Holding (BH-1400)	4:30	64	100%	2 × 2 × 2	112 × 112	1400	30	65	4	6/8	
pCASL	5:15	24	100%	3.4 × 3.4 × 4.2	64 × 64	3800	17	90	N/A	7/8	Label offset = 80 mm, post label delay = 1000 ms
T2 SPACE	3:52	176	100%	1 × 1 × 1	256 × 256	3200	305	varies	N/A	off	

MPRAGE = Magnetization Prepared Rapid Gradient Echo; SPACE = Sampling Perfection, Application Optimized Contrast; FLAIR = Fluid-Attenuated Inversion Recovery; dMRI = diffusion MRI; R-fMRI = Resting state functional MRI; mb = Multiband; sb = Singleband; pCASL = Pseudo-Continuous Arterial Spin Labeling; FOV = Field of View; TR = Repetition time; TE = Echo time; TI = Inversion time.

#### Physical Health Assessments

All NKI-RS sub studies followed the same procedures for assessing height, weight, waist/hip circumference, heart rate, blood pressure, grip strength, vision, and activity levels/sleep. Routine venipuncture assays were acquired at each visit as part of the physical health protocol for laboratory analysis and to contribute to the biospecimen bank at the NIMH Genetics Repository. See^4^ for a detailed description of those procedures.

The MIVAC sub study assessed participant cardiorespiratory fitness and walking speed at baseline and each follow-up visit. Trained study research technicians, under the supervision of MPI Colcombe, administered gold standard submaximal VO_2_ assessments using a recumbent stationary bike connected to the Parvo Medics True One 2400 metabolic measurement system^29^. During the testing session, participants wore a fitted silicon mask attached to the metabolic measurement system, quantifying the relative concentrations of oxygen and CO_2_ in their exhaled breath gasses, as well as a sensor to measure heart rate. Participants pedaled with the aim of reaching at least 90% of their age-predicted maximum heart rate (220-age) and achieving a respiratory exchange ratio (RER; CO_2_ to O_2_ ratio) of 1.02 or higher. During administration, medical support was provided by on-site clinicians. We include multiple data quality metrics in our release to facilitate independent data screening approaches (beginning and ending heart rate, RER, total time on VO2 task, reason for VO_2_ testing termination (criteria met, volitional failure, equipment failure, meeting goal of RER > or equal 1.02 and HR > or equal to 90% age adjusted heart rate max (220-age)).

Walking speed alone and under cognitive load was assessed as a low tech but clinically scalable supplement to the gold-standard cardiorespiratory fitness assessment and has been shown to predict falls, disability, and cognitive decline^30^. Using a protocol developed by researchers at the Albert Einstein College of Medicine^31^, participants were instructed to walk at their normal pace along a 20-foot distance marked off with tape, recite alternate letters of the alphabet, and walk while reciting alternate letters of the alphabet. Two trials were completed for each condition. Walking time, total correct letters, and total errors were recorded by a trained research technician for each trial across single and dual task conditions. See https://rocklandsample.org/mivac-full-end-user-protocol-2 for full details of both physical assessments.

## Data Records

### Data Access

The primary website for all NKI-Rockland Initiative studies, including MIVAC, is located at 10.15387/fcp_indi.retro.NKIRockland. Information specific to MIVAC data can be accessed via^32^, which contains detailed documentation, descriptive information, and instructions for downloading the static dataset used in this manuscript.

### Data Access Levels

Recognizing the potential risks associated with sharing phenotypic data which is high dimensional and personal in nature, the NKI-RS provides two access levels:

#### NKI-Rockland Lite Release

The Lite Release provides open access to only de-identified raw imaging data and limited phenotyping (e.g. age [integer in years], sex, handedness). We defaced MRI structural scans, converted DICOM files to NIfTI format, and stripped all personally identifiable information (PII) from the metadata headers. The NKI-Rockland Lite is immediately available without Data Usage Agreement (DUA) requirements. See “Downloading imaging data” below for details on access.

#### NKI-Rockland Full Phenotypic Release

This Full Phenotypic Release provides access to the full high-dimensional phenotypic protocol common across all NKI-RS sub studies. Users of the NKI-Rockland Full Phenotypic Release complete a data usage agreement (DUA) and obtain appropriate institutional approval prior to accessing data (See https://rocklandsample.org/phenotypic-data for instructions). The intent of the DUA is to ensure that data users (1) agree to protect participant confidentiality when handling the high dimensional NKI-RS phenotypic data and (2) agree to take the necessary measures to prevent breaches of privacy. The DUA does not place any constraints on the range of analyses that can be carried out using shared data, nor does it include requirements for review or co-authorship by the originators of the NKI-RS. As of July 2025, 349 collaborative research sites have obtained full access to the enhanced NKI-RS dataset by signing a DUA. Prior to data sharing, datasets were de-identified by removing visit dates and other personal identifiers. Additionally, open-ended text responses were scrubbed to ensure participant anonymity.

#### Downloading imaging data and the Lite Release

All raw, deidentified imaging data can be accessed through the Rockland Sample portal (https://rocklandsample.org/accessing-the-neuroimaging-data-releases). This website provides instructions for users to directly download data from an Amazon Simple Storage Service (S3) bucket. These data are organized in the Brain Imaging Data Structure (BIDS) format. We provide two scripts to perform batch download of these imaging files, written in Bash and Python. The Python script has the capability to perform basic filtering prior to downloading data. The available filters are: sex, age, session, and image type. Instructions are also provided to download individual files using open-source S3 bucket browsers.

#### Downloading phenotypic data

The latest version of the full NKI-RS phenotypic dataset is available to approved users via the Longitudinal Online Research and Imaging System^33^ (LORIS) database and maintained by NKI-RS program staff as a curated resource. This webpage (https://rocklandsample.org/phenotypic-data) contains instructions for accessing the phenotypic data.

Archived versions of the dataset are available through LORIS as a zipped folder of comma-separated values (CSV) files. Individual versions with cumulative changelogs will be listed in https://rocklandsample.org/archive_static_datasets. Instructions for accessing an archived static version of the dataset used to generate this data paper are available here: 10.15387/MIVAC.

### Description of Dataset

All phenotypic data are available as csv files in long format (each session data in separate rows) with custom_ID linking session-specific participant data across instruments. There are 77 instruments available for MIVAC phenotypic data; file names and column headings are described in detail in the data dictionaries available for download at https://rocklandsample.org/wp-content/uploads/2026/03/Rockland-Sample-LORIS-data-dictionaries.zip. Variables presented in this manuscript are listed in a table downloadable from 10.15387/MIVAC, along with associated scripts used to calculate some of the variables.

Imaging data are organized in the Brain Imaging Data Structure (BIDS) as nifti (.nii) files. Physiological and basic demographics information for imaging data, per BIDS structure requirement, are saved as tsv files with the same file name as the corresponding nii files.

### Partial and missing data

Some participants were not able to successfully complete all components of the NKI-RS protocol due to a variety of factors (e.g., unexpected discomfort in the scanner, family emergency interrupting a testing day). To prevent data loss due to participant discomfort during an MRI session, we included a mock MRI scanner experience during the first visit, when possible. Overall, we attempted to collect as much data as possible within the allotted data collection intervals and logged data losses as they occurred.

### Data license

All NKI-Rockland Sample data are distributed using the Creative Commons-Attribution-Noncommercial license, which is described at: https://creativecommons.org/licenses/by-nc/4.0/legalcode. For the high-dimensional phenotypic data, all terms specified by the DUA must be complied with.

## Technical Validation

### Sample Composition

#### Geographic Distribution

**Fig. 3:**
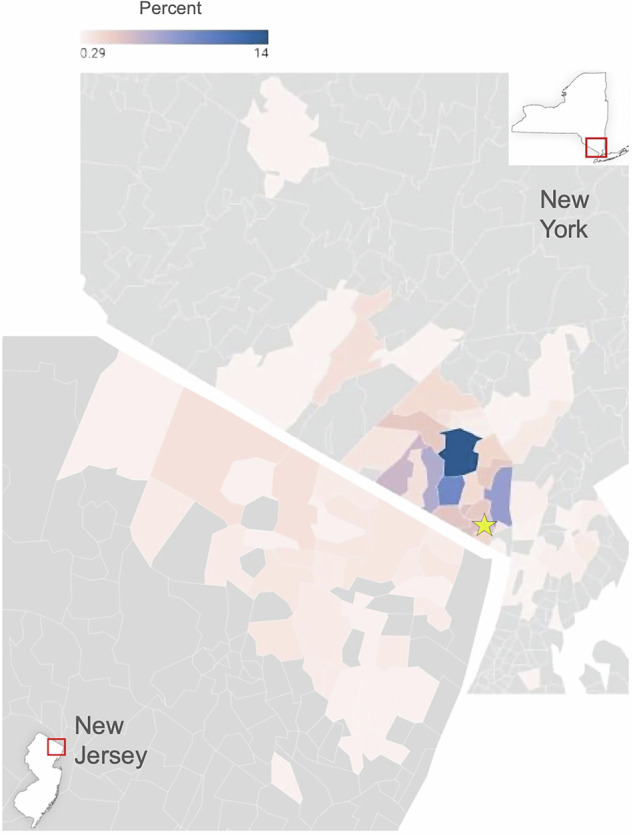
Geographic distribution of participants by zipcode. Color indicates percentages of participants in each zip code. Star indicates location of the Nathan Kline Institute.

The Nathan Kline Institute (NKI) is located 25 miles north of New York City in Rockland County. From 2013 through 2017, the NKI-RS initiative enrolled residents of Rockland and neighboring counties (Bergen County, NJ; Orange County, NY; and Westchester County, NY). A previously published report compared the aggregate NKI-RS dataset (n = 1,610) with the demographics of Rockland County, New York State, and the United States based on census data and demonstrated similar distributions across the research sample, local, regional, and national demographics in most domains^4^. Here we present the MIVAC cohort compared with US census data for additional comparison (Table 4). In the current study, similar to reporting on the aggregate dataset, individuals who lived closer to NKI were more likely to enroll. See Fig. 3.

**Table 4:** Demographics of the NKI-RS MIVAC sub study (NKI-RS enrollment 2011–2017) and United States Census Data (2009).

	NKI-RS MIVAC	USA
**Population (estimate)**	348	309,529,237
**Persons 65 years old and over**	22.70%	13%
**Female persons**	71.30%	51%
**White**	83.30%	79.60%
**Black or African American**	10.10%	13%
**American Indian/Alaska Native**	0.60%	1%
**Asian**	4.60%	5%
**Native Hawaiian or Other Pacific Islander**	0.30%	0%
**Two or more races**	N/A	2%
**Hispanic or Latinx**	8.00%	16%
**Foreign born**	N/A	5%
**Bilingual at home**	27.16%	18%
**High school graduates**	98.80%	80%
**Bachelor’s degree or higher**	69.30%	24%
**Persons per household**	2.9	2.51
**Median household income**	$75,000 - $99,900	$52,029
**Per capita money income**	N/A	$21,587
**Persons below poverty level**	N/A	12.20%

#### Age, Sex, and Clinical Characterization

Age distributions (Fig. 1b) were relatively even across the middle to older adult age range, despite known challenges recruiting working age adults into time intensive research protocols. The contemporaneous recruitment effort for the pediatric longitudinal sub study allowed parents to learn about the MIVAC study and gain direct experience with the research staff, facilitating enrollment across both sub studies. Of the 348 participants who completed baseline assessments, 248 (71%) were female. Midlife and older adult participation among men tends to lag behind female participation in studies of healthy aging^34^. Cross enrollment with the pediatric sub study may have further contributed to this gender gap, as mothers were often the accompanying guardian.

Clinical characterization of global cognition was conducted as a brief screening using the MoCA. Across 808 total assessments, there were 543 scores above 25 (67%), 259 scores between 25 and 18 (32%), and 6 scores below 18 (.7%). Based on the study neuropsychologist review, there was no supporting evidence for dementia among participants who scored 25 or lower, including the 4 participants contributing 6 scores below 18. According to standard MoCA test score criteria^28^, 223 (65%) participants were characterized as cognitively normal at baseline (MoCA > 25), and 120 (35%) participants scored in the range suggestive of mild or moderate cognitive impairment at baseline. No study participants scored in the severe range (0–9). Of the 257 participants with baseline and at least one follow-up visit, change of classification (i.e., MoCA classification based on test performance) was observed in 27% of participants, 28 (11%) fell from “normal” to “mild,” 39 (15%) rose from “mild” to “normal” classification, and 3 (.01%) rose from “moderate” to “mild.” All 4 participants who scored in the “moderate” range (10–17) scored higher at their final follow-up visit. Please note, MoCA score labels are assigned based on single score cutoffs established by^28^ and are not a diagnostic determination.

**Fig. 4:**
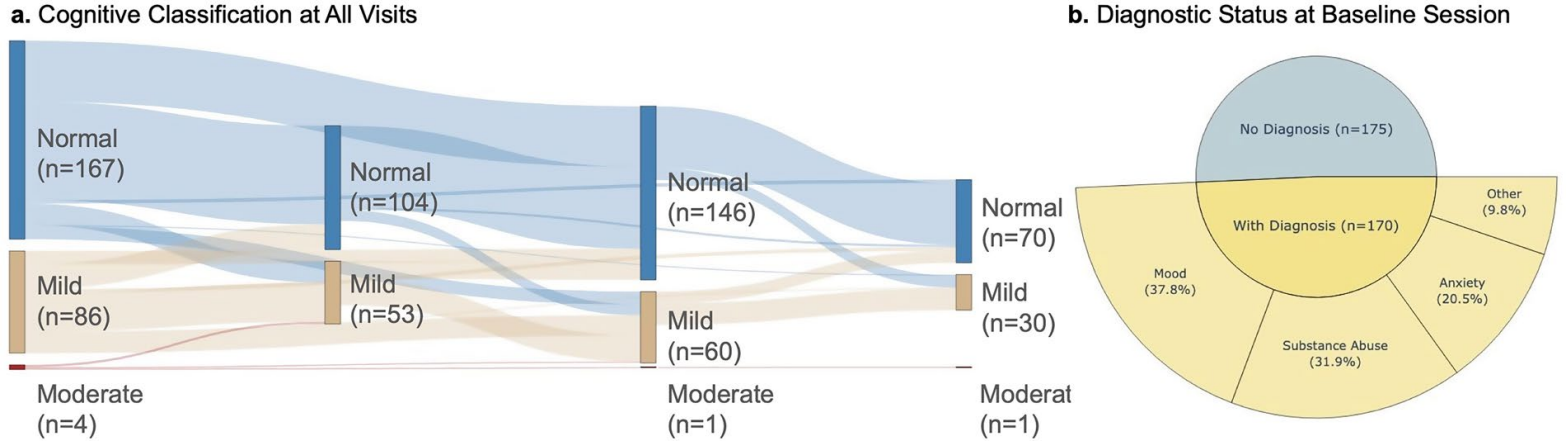
Classifications of cognition and major psychiatric categories. (**a**) Cognitive classification of ‘Normal’, ‘Mild’ (impairment), and ‘Moderate’ (impairment) refer to the standard MoCA nomenclature. Cognitive classification stability and change across four study timepoints based on MoCA test score criteria are shown for all participants with baseline and at least one follow-up visit (n = 257: 6 participants did not have a MoCA score at baseline; 85 participants did not have a MoCA follow-up score). Scores at baseline (left) are compared with all available follow-up sessions for each participant. (**b**) DSM-IV diagnostic status at baseline (via Structured Clinical Interview for DSM-IV-TR Axis I Disorders – Non-Patient Edition (SCID-I/NP), n = 345, 3 participants did not complete a clinical interview) and the relative percent of mood, substance, anxiety, and other disorders. Includes past and present DSM-IV diagnoses. “Other” diagnoses included: Hypochondriasis: n = 2; Anorexia Nervosa: n = 5; Eating Disorder NOS: n = 6; Bulimia Nervosa: n = 3; Attention-Deficit/Hyperactivity Disorder: n = 2; ADHD, Predominantly Inattentive Type: n = 2; ADHD, Combined Type: n = 1; ADHD NOS: n = 1; Bereavement: n = 4. Multiple diagnoses per participant were low (two = 42, three = 19, four or more = 17) and not included in the figure.

See Fig. 4a for an illustration of MoCA score classification change from baseline to final follow-up visit. In a comparison of the subset of participants with MoCA score change in the negative direction (n = 87) to those with improved (n = 131) or stable (n = 39) scores, there were no differences in age (mean (sd) - 56.59 (8.89) vs 56.10 (9.68) vs 56.62 (10.05), respectively) or level of education (mean (sd) - 15.89 (2.25) vs 15.86 (2.11) vs 16.77 (2.52), respectively).

Psychiatric characterization via semi-structured SCID-I/NP^35^ interviews determined approximately half of the participants (n = 172, 51%) had no DSM-IV diagnosis at baseline. Among those with a diagnosis (n = 169, 49%), major diagnostic categories were relatively evenly split among mood (38%), substance abuse (32%), and anxiety (20%). There was a relatively low frequency of other disorders (10%) and multiple diagnoses per participant (21% with three or more diagnoses). See Fig. 4b for more detail.

### Quality assessment

#### Phenotypic data

Prior to each NKI-RS release, scatterplots by age and boxplots comparing past releases to each progressive release were reviewed. Phenotypic data were not “cleaned” of outliers unless overt technical errors were identified in pre-release reviews. Data review and cleaning is expected to be incorporated into each independent research analysis.

**Fig. 5:**
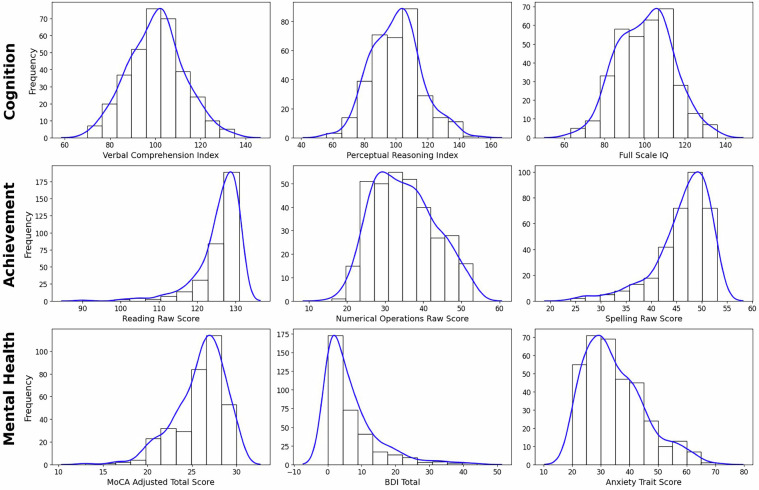
Distributions of cognitive abilities, academic achievement, and mental health measures.*Top row*: composite scores from WASI-II. *Middle row*: raw scores from WIAT. *Bottom row left to right*: MoCA education-adjusted total score, BDI total score, and STAI raw Anxiety trait score. Only baseline data are included and no filtering was done.

**Fig. 6:**
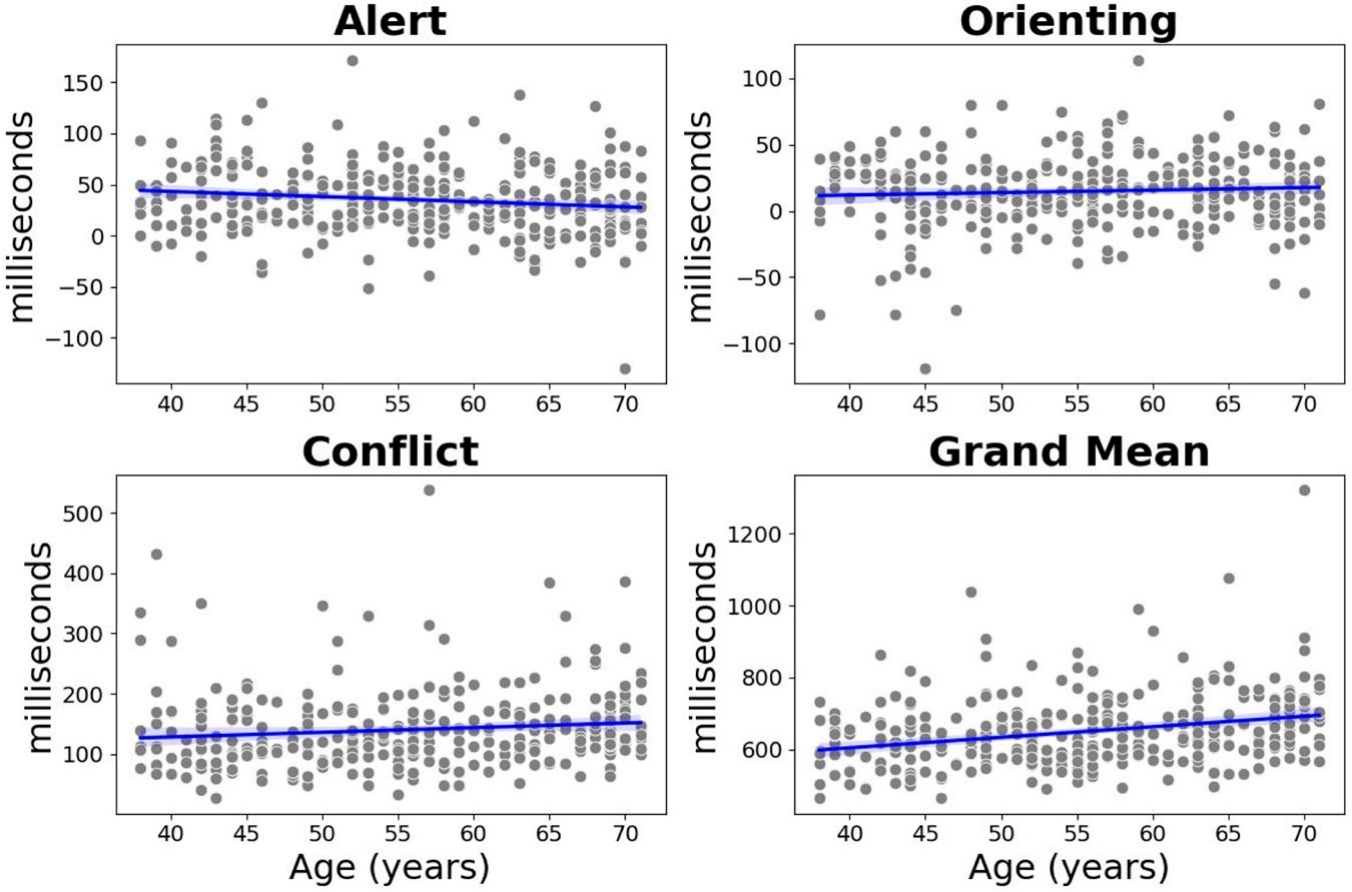
Scatter plots of Attention Network Task subscores and grand mean against age. Alert: t(332) = −2.73, p = 0.007; Orienting: t(332) = 1.23, p = 0.220; Conflict: t(330) = 2.12, p = 0.034; Grand Mean: t(330) = 5.33, p < 0.001. Plots include all unfiltered baseline data.

**Fig. 7:**
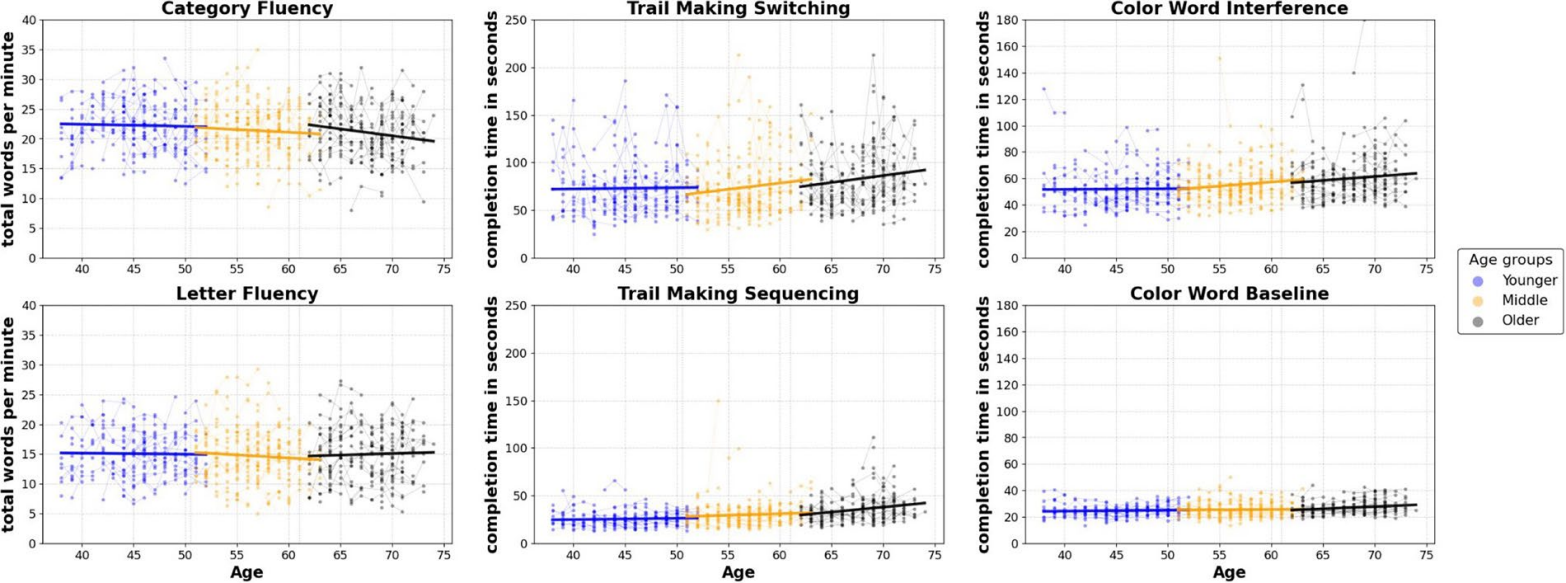
Delis-Kaplan Executive Function Scale Subtest Scores across longitudinal timepoints, color coded for three age tertile groups (Younger (blue): <  = 50.5; Middle (orange): > 50.5 and <  = 61; Older (black): > 61). Left panel shows performance on category and letter fluency (CF, LF). Middle panel shows Trail Making Test switching and sequencing conditions (Switch, SeQ). Right panel shows Color Word Interference Test interference and averaged baseline conditions (INT, BASE). Three bolded lines are regression lines for the three age groups in each plot. Significant age effects were observed for: CF Older: t(256) = −2.60, p = 0.010; Switch Middle: t(263) = 2.10, p = 0.037; Switch Older: t(263) = 2.43, p = 0.016; SeQ Older: t(280) = 4.15, p < 0.001; INT Middle: t(268) = 2.23, p = 0.026; BASE Older: t(270) = 3.33, p = 0.001.

Figures 5–7 summarize score distributions and age-associated performance trends for core cognitive and mental health measures. Figure 5 illustrates both roughly symmetric (e.g., WASI-II index scores, WIAT-II Numerical Operations) and skewed distributions (e.g., WIAT-II Reading and Spelling, MoCA, BDI, STAI). Figure 6 shows modest but statistically significant age effects for the Attention Network Task in alerting, conflict, and grand mean reaction time. Figure 7 (top row) demonstrates age-related performance declines on D-KEFS Category Fluency, Trail Making Test–Switching, and Color Word Interference Test - Inhibition. Figure 7 (bottom row) highlights relatively stable performance across age for Letter Fluency, Trail Making Test -Sequencing, and a composite score derived from the baseline conditions of the Color Word Interference Test (Color Naming and Word Reading). Greater variability in performance was observed for conditions with longer completion times (Trail Making Test-Switching, Color Word Interference Test-Inhibition) and a greater number of total words generated (Category Fluency). Cognitive performance for this sample is within normative expectations as evidenced by WASI-II index scores (mean = 100, standard deviation = 15) centered at 100. D-KEFS scaled scores (mean = 10, standard deviation = 3) were also in the average range (scaled scores ranged from 10-12) across the subtests reported in Fig. 7. Note: D-KEFS raw scores, not scaled scores, are shown in Fig. 7.

**Fig. 8:**
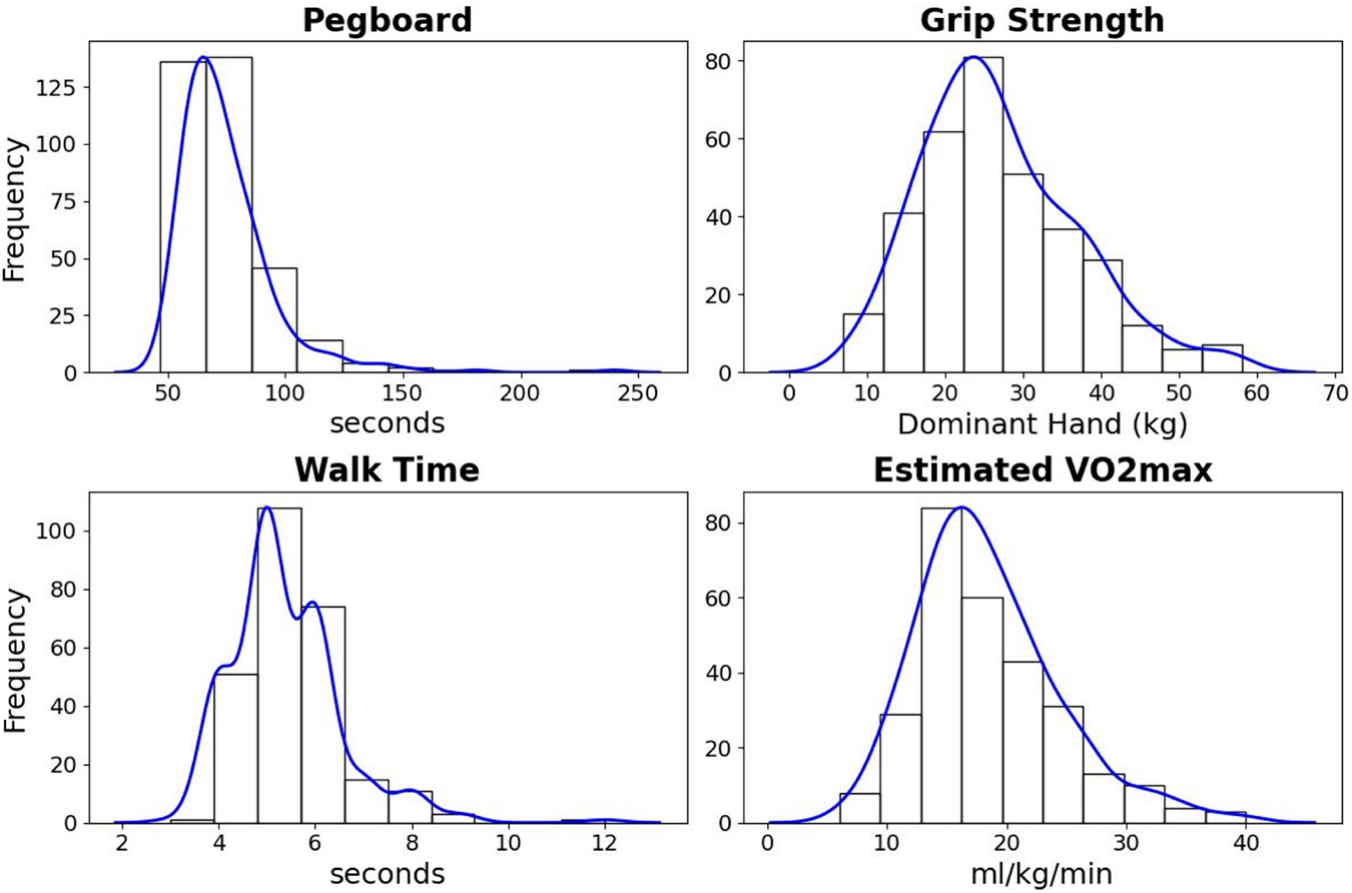
Distributions of physical measures: *Top: left* - Grooved pegboard completion time for dominant hand, *right* - Grip strength for dominant hand. *Bottom*: *left* - Single task walking time from Walking While Talking Task, *right* - Estimated VO_2_max from submaximal cardiorespiratory fitness test. Only baseline data are included and no filtering was done.

Figure 8 presents the histogram and kernel density estimate for distributions of physical health measures, including grooved pegboard, grip strength, walk time, and VO₂ max. Performance was relatively lower across all physical health measures compared to approximately age-matched comparison groups^36–39^. For example, the mean age and sex-corrected percentile scores for cycle ergometry-estimated VO_2_max (FRIEND Registry^39^) places our sample at the 44^th^ percentile, modestly lower than average (50^th^ percentile).

**Fig. 9:**
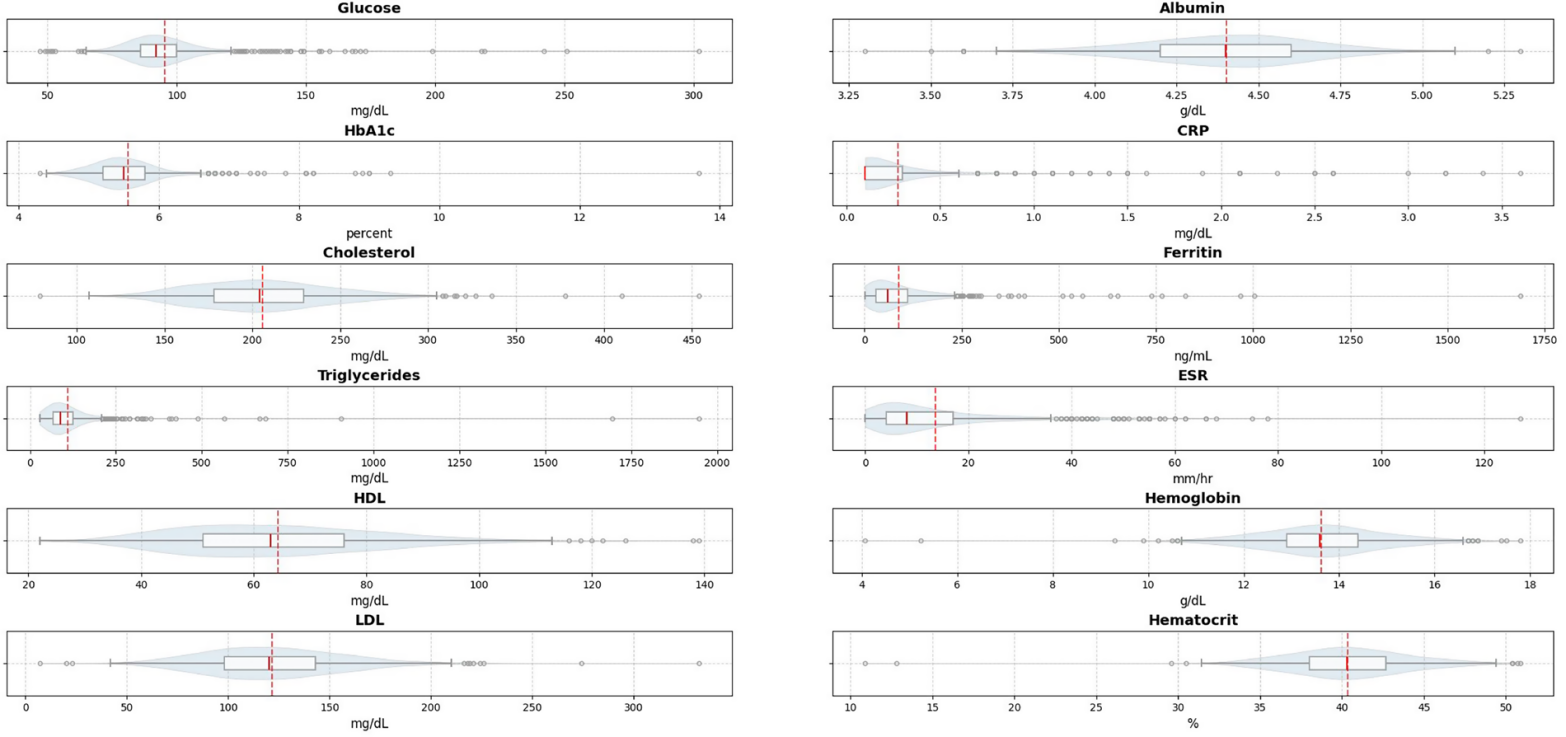
Boxplots with kernel-density overlays for each analyte from the blood sample collection. HbA1c = Hemoglobin A1C; HDL = High-density lipoprotein; LDL = Low-density lipoprotein; CRP = C-reactive protein; ESR = Erythrocyte sedimentation rate.

Figure 9 depicts boxplots with kernel-density overlays for each analyte from the blood sample collection. Distributions were largely unimodal and centered within expected adult ranges, with several measures showing right-tailed skew typical of metabolic/inflammatory indices. Extreme values are visible in the tails; consistent with our data policy, these observations were retained (no outlier trimming) to maximize transparency and enable users to apply domain-appropriate handling (e.g., robust estimators) in downstream analyses. Missingness was modest and chiefly attributable to occasional deferred phlebotomy or scheduling constraints. See Table 5 for a summary by assay (sample sizes, median, IQR, 5th–95th percentiles). Whole blood and cell line samples are available by request to the NIMH Repository and Genomics Resource.

**Table 5:** Baseline descriptors for each analyte from the blood sample collection.

Lab	n	Median	IQR	[5, 95%ile]
Glucose	279	92	15	[77.9, 122.3]
HbA1c	274	5.4	0.5	[4.9, 6.535]
Cholesterol	279	208	49.5	[150, 274]
Triglyceride	279	93	59	[45, 210.3]
HDL	275	63	27.5	[38.7, 97]
LDL	273	121	51	[70.6, 179.4]
Albumin	279	4.5	0.3	[4, 4.81]
CRP	276	0.1	0.2	[0.1, 0.8]
Ferritin	275	64	91	[9, 217.6]
ESR	278	8	12	[2, 45.45]
Hemoglobin	278	13.6	1.6	[11.49, 15.82]
Hematocrit	278	40.6	4.675	[34.89, 46.82]

HbA1c = Hemoglobin A1C; HDL = High-density lipoprotein; LDL = Low-density lipoprotein; CRP = C-reactive protein; ESR = Erythrocyte sedimentation rate.

To evaluate the long term stability of key phenotypic measures over time, intraclass correlation coefficients (ICCs) were calculated using participants with at least three annual longitudinal assessments. Cognitive indices from the WASI-II showed good stability, with agreement/consistency values ranging from .67/.71 (Similarities) to .77/.77 (Matrix Reasoning) to .79/.83 (Vocabulary) to .90/.90 (Block Design). The MoCA demonstrated moderate stability (.63/.64), while affective measures were higher (Beck Depression Inventory; BDI = .75/.75; State Trait Anxiety Inventory-Trait; STAI-T = .81/.81). Practice-related gains were observed for the WASI-II and MoCA, whereas BDI and STAI-T scores remained stable (See Table 6 for details here and below). Estimates for the Attention Network Task, consistent with prior reports^40^, showed moderate test-retest stability for conflict (.48/.50) and grand mean reaction time (.66/.67), but lower values for alerting (.38/.39) and orienting (.16/.17). Practice effects included increases in alerting and orienting effects, decreases in conflict effects, and faster overall response times across sessions. D-KEFS tasks demonstrated moderate to high retest stability, with Color Word Interference scores ranging from .85 to .87 across baseline and interference conditions. Trail Making Test sequencing conditions showed moderate retest stability (number = 0.57/ 0.58; letter = 0.64/ 0.65), while the switching condition was higher (.74/.75) and most sensitive to practice effects, t(196) = −2.78, p = 0.006. Tower total achievement score showed moderate retest stability (0.53/ 0.55) with improved scores over time (t(197) = 6.88, p < 0.0001), and verbal fluency measures were higher (letter = 0.83/ 0.84; category = 0.72/ 0.73) with increasing word production across sessions (t(177) = 4.27, p < 0.001 and t(178) = 2.60, p = 0.01 for letter and category, respectively). Motor praxis reaction time, conditional exclusion test reaction time, two-back total correct, and continuous performance test d-prime from the Penn Computerized Neurocognitive Battery (Penn CNB)^41^ ranged in test-retest stability from a low of .47 consistency for two-back to a high of 0.74 agreement for the continuous performance test. Performance gains over annual follow-up visits were observed for motor praxis, two-back, and the continuous performance test, but not for reaction time on the conditional exclusion test (see Table 6). Physical measures ranged from relatively high test-retest stability (grip strength = .78/.78; VO₂ max = .83/.83) to moderate (pegboard completion = .68/.69; walk time = .46/.47). There were no observed practice effects for grip strength or VO₂ max, while pegboard times decreased with repeated testing and walk times increased. Overall, most measures demonstrated moderate to high longitudinal stability, with practice effects most pronounced in cognitive and motor speed tasks. ICCs and mean slopes for a sampling of the measures are reported in detail in Table 6.

**Table 6:** Intraclass Correlation Coefficients (ICCs) for phenotypic measures.

Variable	n	ICC agreement	agreement 95% CI	ICC consistency	consistency 95% CI	cohen’s d slope	t	p
WASI BD	198	0.90	[0.87, 0.92]	0.90	[0.88, 0.92]	0.22	3.05	0.0026
WASI VOC	198	0.79	[0.75, 0.83]	0.83	[0.79, 0.86]	0.71	9.94	< 0.0001
WASI MR	198	0.77	[0.72, 0.81]	0.77	[0.72, 0.82]	0.21	2.98	0.0033
WASI SIM	197	0.67	[0.61, 0.73]	0.71	[0.65, 0.76]	0.53	7.48	< 0.0001
MoCA	115	0.63	[0.54, 0.72]	0.64	[0.55, 0.73]	0.33	3.51	0.0006
RAVLT	177	0.72	[0.66, 0.77]	0.76	[0.71, 0.81]	0.70	9.25	< 0.0001
DKEFS CF	179	0.72	[0.66, 0.78]	0.73	[0.67, 0.78]	0.19	2.59	0.0103
DKEFS TMT	197	0.74	[0.69, 0.79]	0.75	[0.69, 0.80]	−0.20	−2.78	0.006
ANT Alert	192	0.38	[0.30, 0.47]	0.39	[0.30, 0.48]	0.27	3.74	0.0002
ANT Orienting	191	0.16	[0.08, 0.26]	0.17	[0.08, 0.26]	0.20	2.70	0.0076
ANT Conflict	190	0.48	[0.40, 0.56]	0.5	[0.42, 0.58]	−0.38	−5.21	< 0.0001
ANT Grd Mean	190	0.66	[0.59, 0.72]	0.67	[0.61, 0.73]	−0.34	−4.70	< 0.0001
MPraxis	170	0.72	[0.66, 0.78]	0.72	[0.66, 0.78]	0.18	2.35	0.0197
CET	170	0.74	[0.68, 0.80]	0.74	[0.68, 0.79]	0.05	0.65	0.5155
2Back	170	0.47	[0.38, 0.56]	0.47	[0.38, 0.56]	0.14	1.80	0.073
CPT	171	0.49	[0.40, 0.57]	0.51	[0.42, 0.59]	0.42	5.49	< 0.0001
BMI	204	0.95	[0.93, 0.96]	0.95	[0.93, 0.96]	−0.06	−0.81	0.4186
HbA1c	132	0.74	[0.67, 0.80]	0.74	[0.67, 0.80]	0.39	4.46	< 0.0001
Cholesterol	137	0.64	[0.56, 0.71]	0.65	[0.57, 0.72]	−0.25	−2.89	0.0045
Pegboard	200	0.68	[0.62, 0.74]	0.69	[0.63, 0.75]	−0.26	−3.70	0.0003
Grip	196	0.78	[0.73, 0.82]	0.78	[0.73, 0.82]	−0.07	−1.01	0.3129
WWT WalkTime	154	0.46	[0.36, 0.55]	0.47	[0.38, 0.57]	0.28	3.41	0.0008
VO2max	174	0.83	[0.79, 0.87]	0.83	[0.79, 0.87]	−0.04	−0.58	0.5632
BDI	199	0.75	[0.69, 0.79]	0.75	[0.69, 0.79]	−0.08	−1.07	0.2858
STAI	203	0.81	[0.76, 0.84]	0.81	[0.76, 0.84]	−0.09	−1.35	0.1787
PSQI	133	0.64	[0.55, 0.71]	0.64	[0.55, 0.72]	−0.01	−0.16	0.8712
CogFailureQuest	157	0.79	[0.74, 0.84]	0.81	[0.76, 0.85]	−0.37	−4.68	< 0.0001
SocialNetwork	155	0.64	[0.56, 0.71]	0.64	[0.57, 0.71]	−0.06	−0.79	0.4326

**Fig. 10:**
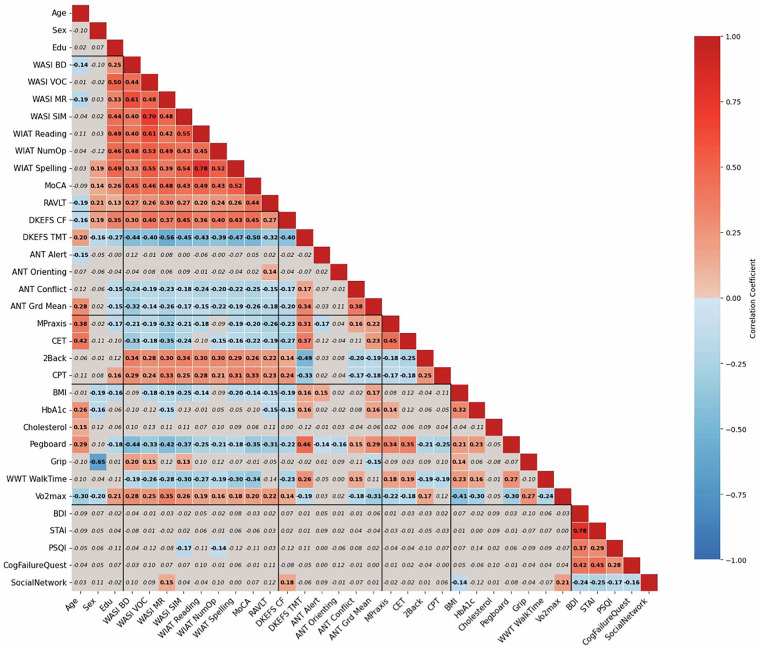
Heat map showing r values for bivariate correlations among demographic and phenotypic measures. Correlations that survived multiple-comparisons correction at p < 0.05 using False-Discovery Rate (Benjamini, Y., & Hochberg, Y. 1995) are shown in colored boxes and bolded font. Non-significant correlations are shown as grey boxes. Four correlations with the most missing data were: WWT WalkTime & HbA1c (missing n = 136), WWT WalkTime & Cholesterol (missing n = 132), HbA1c & VO_2_max (missing n = 118), and Cholesterol & VO_2_max (missing n = 113). *WASI BD*: Wechsler Abbreviated Scale of Intelligence Block Design raw score; *WASI VOC*: Vocabulary raw score; *WASI MR*: Matrix Reasoning raw score; *WASI SIM:* Similarities raw score; *WIAT Reading*: Wechsler Individual Achievement Test Word Reading raw score; *WIAT NumOP*: Numerical Operations raw score; *WIAT Spelling*: Spelling raw score; *MoCA*: Montreal Cognitive Assessment total score adjusted for education; *RAVLT:* Rey Auditory Verbal Learning Test - long-delay free recall total correct; *DKEFS CF*: Delis-Kaplan Executive Function System Category Fluency raw score; *DKEFS TMT*: Trail Making Test Switch Condition completion time; *ANT Grd Mean*: Attentional Network Test - Grand Mean; *MPraxis*: PENN Computerized Neurobehavioral Battery (CNB) - Motor Praxis reaction time; *CET*: PENN CNB Conditional Exclusion Task reaction time; *2Back*: PENN CNB 2-Back total correct; *CPT*: PENN CNB Continuous Performance Test Number + Letter d-prime; *BMI*: Body Mass Index; *HbA1c*: Hemoglobin A1c; *Grip*: Grip Strength in kg for dominant hand; *WWT WalkTime*: Walking While Talking - first trial of single task walking time; *VO*_*2*_*max*: estimated from the submaximal test; *BDI*: Beck Depression Inventory total; *STAI*: State-Trait Anxiety Inventory - total trait raw score; *PSQI*: Pittsburgh Sleep Quality Index total sleep score; *CogFailureQuest*: Cognitive Failures Questionnaire total score; *Social Network*: Social Network Size. Only baseline data are included and no filtering was done.

CI = Confidence Intervals; See Fig. 10 for definitions of variable abbreviations. Only baseline data are included and no filtering was done.

**Fig. 11:**
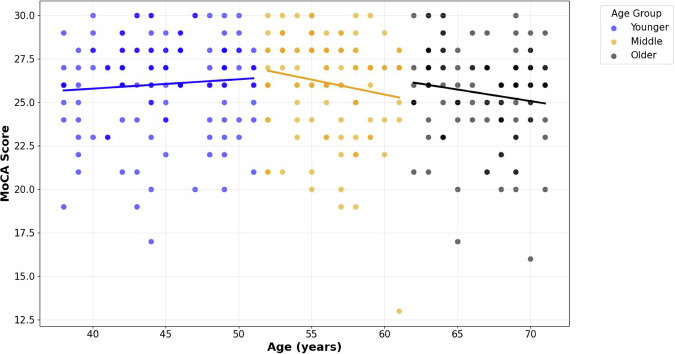
Scatter plot of MoCA Score against Age, divided into three age groups by tertiles (Younger: < / = 51; Middle: > 51 and < / = 61; Older: > 61). The three regression lines are specific to each age group (Younger: n = 121, t = 0.83, p = 0.41; Middle: n = 110, t = −1.66, p = 0.10; Older: n = 112, t = −1.49, p = 0.14. All unfiltered baseline data were included in the plot.

**Fig. 12:**
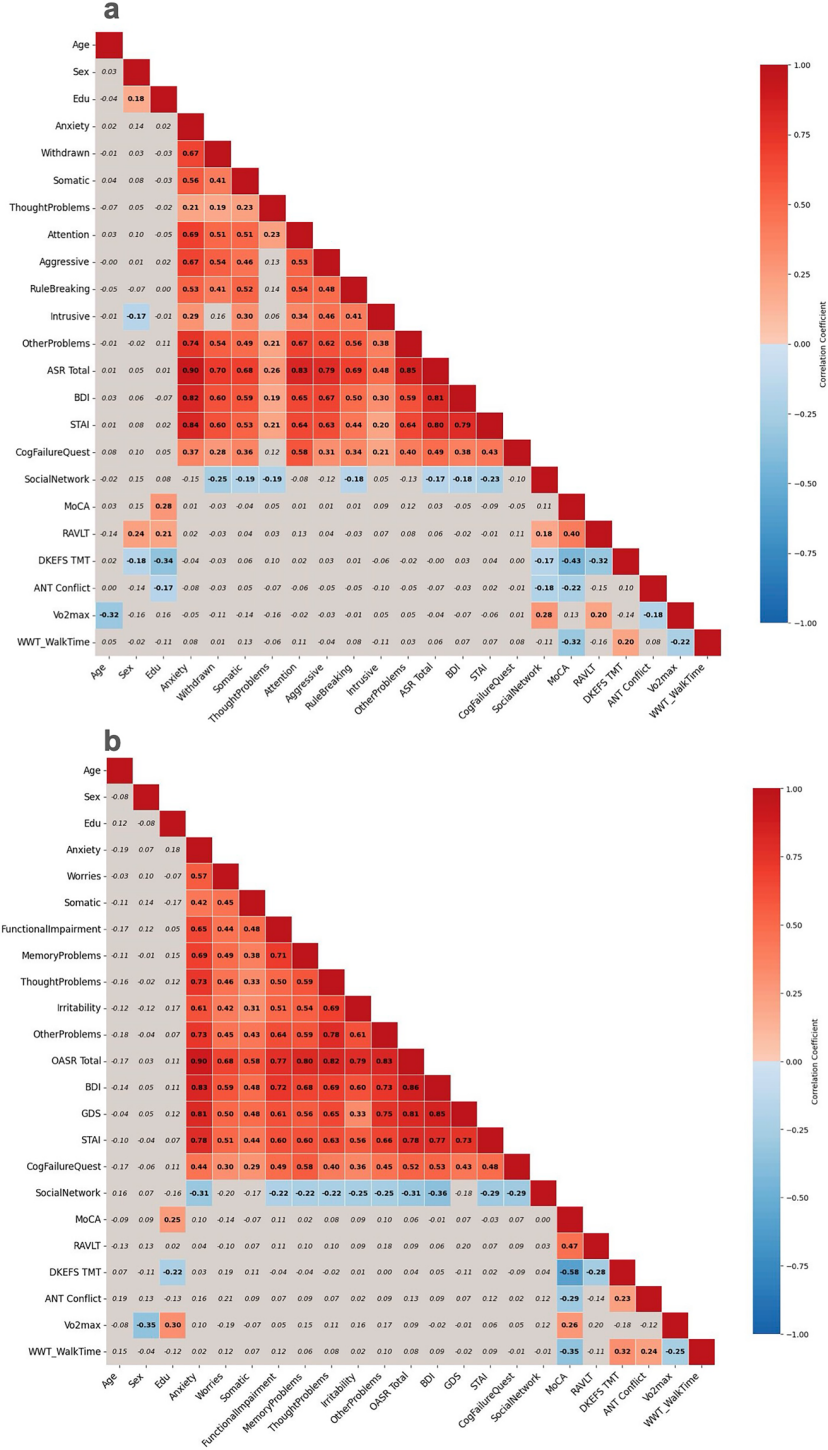
Heat map showing coefficients for bivariate correlations among demographic and subset of phenotypic measures against subscales of: (**a**) Adult Self Report (ASR; administered to participants < 60 years old) and (**b**) Older Adult Self Report (OASR; administered to participants 60+ years old) and total scores. Values that survived False-Discovery Rate multiple-comparisons correction at p < 0.05 are shown in bolded font and colored boxes. *BDI*: Beck Depression Inventory total; *GDS*: Geriatric Depression Scale; *STAI*: State-Trait Anxiety Inventory - total trait raw score; *CogFailureQuest*: Cognitive Failures Questionnaire total score; *Social Network*: Social Network Size, *MoCA*: Montreal Cognitive Assessment total score adjusted for education; *RAVLT*: Rey Auditory Verbal Learning Test - long delay free recall total correct; *DKEFS TMT:* Delis-Kaplan Executive Function System Trail Making Test Switch Condition completion time; *ANT*: Attention Network Test; *VO2max*: estimated from the submaximal test; *WWT WalkTime*: Walking While Talking - first trial of single task walking time. Only baseline data are included and no filtering was done.

We next examined correlations among phenotypic variables to facilitate evaluation of measures (Fig. 10). All correlations were corrected for multiple-comparisons with False Discovery Rate^42^. To ensure transparency, outliers were not removed for this descriptor, though we recommend careful data review and outlier handling in subsequent analyses. Broad patterns of association across cognitive, health, and demographic domains were consistent with established findings^43,44^. For example, (1) age correlated with fluid (BD, MR) but not crystallized (VOC, SIM) WASI-II subtests^45–47^, was negatively associated with RAVLT delayed recall^48,49^ and category fluency^50,51^, and positively associated with Trail Making Test completion time^51–53^; (2) age was related to poorer physical but not mental health^54–56^; (3) self-reported mood, sleep, and subjective cognitive impairment were intercorrelated^57,58^; (4) the Attention Network Task conflict effect and grand mean response times correlated with other cognitive measures, whereas alerting and orienting did not^59^; (5) the MoCA was associated with episodic memory^60^, executive function^61^, performance on WASI-II subtests^62^, academic achievement^63^, and physical health measures (BMI^64^, pegboard^65^, walk time^30,65–67^, VO_2_max^7^), but not with age^28,68^ or subjective complaints^69^; and (6) VO₂max cardiorespiratory fitness was correlated with all measures of cognition except ANT alerting and orienting^70,71^, social network size^72^, and grip strength^73^, and was negatively associated with BMI^73^ and blood glucose^74^. With respect to MoCA performance and age, some prior reports did not find an association for age in healthy adults between 55 and 85 years old^28^, while others did^75^. Age cohort differences in education level may also be an important factor to consider^76^. See Fig. 11 for MoCA scatterplots by age. See also Fig. 12 for additional correlations among participants’ demographic and phenotypic characteristics split by age 65 and older (OASR) and under 65 (ASR).

#### Neuroimaging data

All NKI-RS imaging datasets are released to users without preprocessing or quality filtering, as there is no universal agreement on quality standards in the field. Additionally, including datasets of varying quality serves two important purposes: it enables researchers to develop new methods for artifact correction and allows for assessment of how real-world data imperfections affect reliability and reproducibility of findings.

#### Structural MRI

**Fig. 13:**
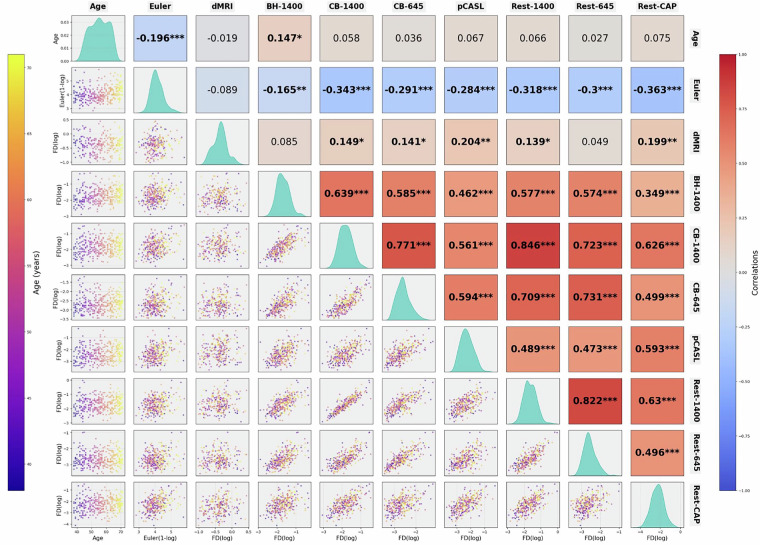
Correlations of mean Framewise Displacement (FD) for MRI sequences with age and FD for other sequences. Lower triangle shows scatterplots with log-transformed FDs, and points were color-coded by participant age. Upper triangle displays the corresponding Pearson correlation coefficients (r) for each correlation, with significance indicated by asterisks (*p < 0.05; **p < 0.01; ***p < 0.001) based on FDR-corrected p-values. Diagonal panels show the distribution of FD values for age and each sequence.

**Fig. 14:**
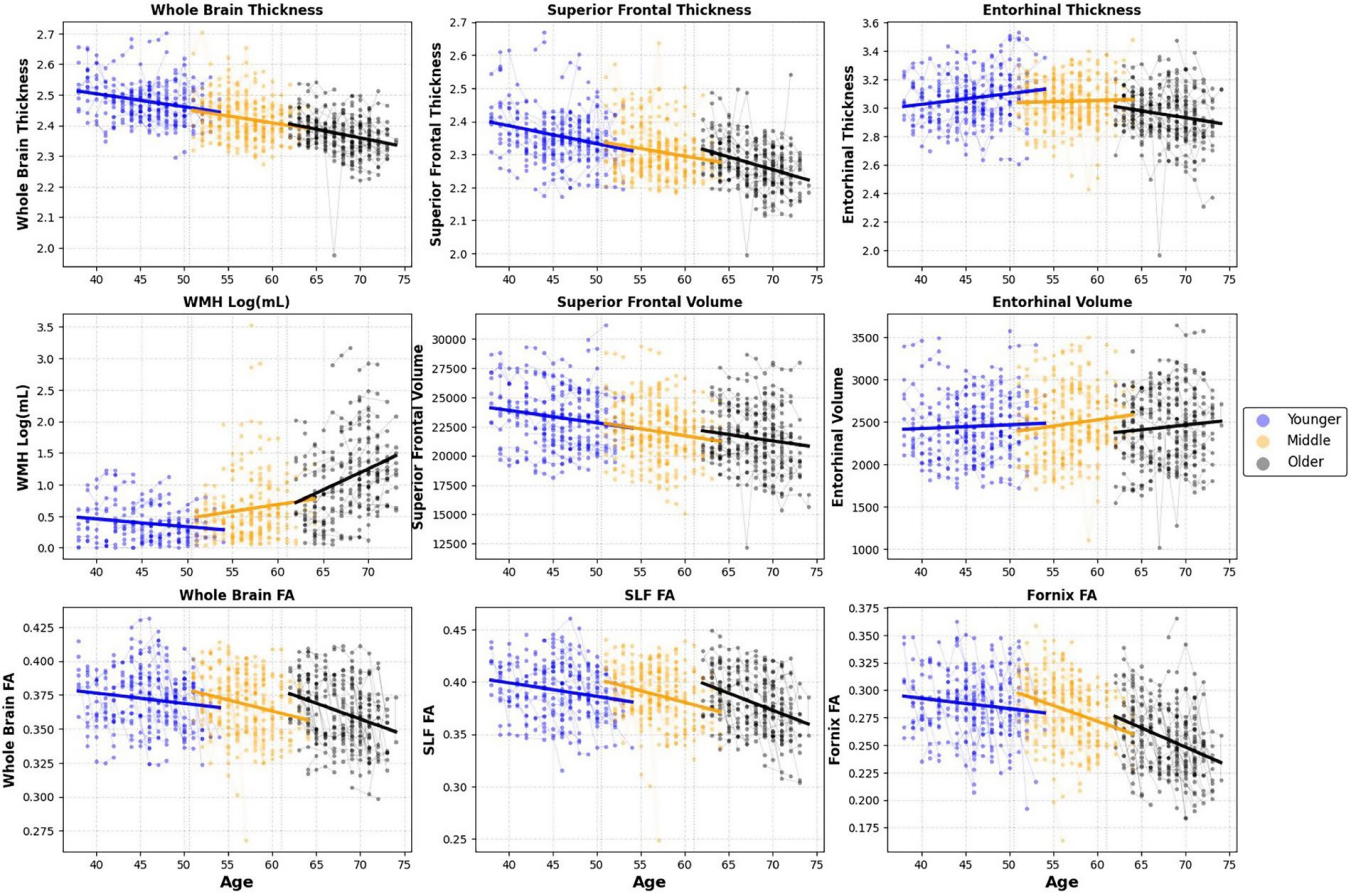
Structural measures from MRI for individual participants collapsing across all timepoints and color coded for three age tertile groups (Younger: <  = 50.5; Middle: > 50.5 and <  = 61; Older: > 61). Row 1 shows the mean cortical thickness for the whole brain and for the Superior Frontal and Entorhinal Cortices (WBt, SFt, ERt). Row 2 shows White Matter Hyperintensity and volumes for the Superior Frontal and Entorhinal Cortices (WMH, SFv, ERv). Row 3 shows the mean fractional anisotropy (FA) across 28 major white matter tracts, for the Superior Longitudinal Fasciculus, and for the Fornix (WBfa, SLFfa, Fxfa). Three bolded lines are age regressions in each plot. Significant age effects were observed for: WBt - Younger: t(282) = −4.25, Middle: t(291) = −3.46, Older: t(313) = −5.09, all p < 0.0001; SFt - Younger: t(282) = −4.34, p < 0.0001, Middle: t(291) = −3.15, p = 0.0018, Older: t(313) = −6.14, p < 0.0001; ERt - Younger: t(282) = 2.81, p = 0.0053, Older: t(313) = −2.91, p = 0.0039; WMH: Younger: t(196) = −2.28, p = 0.0238, Older: t(252) = 5.28, p < 0.0001; SFv: Younger: t(282) = −2.70, p = 0.0074, Middle: t(291) = −2.50, p = .0131, Older: t(313) = −2.25, p = 0.0251; WBfa: Younger: t(263) = −2.16, p = 0.0314, Middle: t(264) = −3.63, p = 0.0003, Older: t(290) = −5.21, p < 0.0001; SLFfa: Younger: t(263) = −3.06, p = 0.0024, Middle: t(264) = −4.25, Older: t(290) = −6.37, both p < 0.0001; Fxfa: Middle: t(263) = −4.92, Older: t(290) = −6.02, both p < 0.0001.

For this technical validation, T1-weighted images were processed with Mindboggle^77^, a software that combines functionalities from Advanced Normalization Tools (ANTS)^78^ and FreeSurfer^79^ to parcellate brain regions using the Desikan-Killiany-Tourville atlas^80^. Euler number was calculated in FreeSurfer to assess data quality, with poorer quality indicated by more negative values^81^. Out of 975 T1 images across all timepoints, 4.0% had Euler numbers more than 2 SD below the mean. As shown in Fig. 13, Euler numbers correlated negatively with age (r = − 0.196, p < 0.001) and, except for dMRI, also correlated with FD from all other sequences (r between − 0.165 to − 0.363, all p < 0.01). Top row of Fig. 14 shows steady decline in the whole brain and superior frontal thickness while entorhinal thickness peaks around middle age followed by gradual decrease into older age, consistent with existing literature.

#### Diffusion MRI

For this technical validation, diffusion MRI data were processed in QSIPrep and QSIRecon^82^. A detailed description of the full pipeline is available on the MIVAC webpage (10.15387/MIVAC). Briefly, QSIPrep performed standard preprocessing using MRtrix3 tools^83^, including denoising, Gibbs unringing, and bias correction. Head motion and Eddy current corrections were then performed with FSL’s eddy tool^84^. Average Framewise Displacement (FD) and age effects are shown in Fig. 13. After preprocessing, QSIRecon’s autotrack pipeline^85^ was used to reconstruct 56 white matter tracts and mean microstructural measures (FA) were calculated for each tract and averaged between the two hemispheres. With a total of 839 dMRI images, 3.34% had FD larger than 2 SD from the mean and 68% of the scans had no bad slices. There was no correlation between age and FD. Age differences in FA for the whole brain and select white matter tracts are presented in row 3 of Fig. 14. Consistent with current literature, FA declines in older age and at the steepest rate in the oldest age group.

#### Functional MRI

Jenkinson’s FD^86^ was calculated for task and resting fMRI scans using AFNI’s 3dvolreg function^87^. Figure 13 shows the cross-correlations among mean FDs for fMRI scans and each with age and other sequences. Among the six fMRI sequences, out of a range of 684 to 696 total images across all timepoints, the percent of data with FD greater than 2 SD above the mean was between 2% to 4%. Among all fMRI sequences, only FD for the Breath-hold sequence with TR of 1400 ms was correlated with age.

Along with other NKI-RS datasets, preprocessed fMRI, T1 and T2-weighted structural MRI images from MIVAC are provided through the Reproducible Brain Charts (RBC) project (https://reprobrainchart.github.io/). Data in RBC was preprocessed using the Configurable Pipeline for the Analysis of Connectomes (C-PAC)^88^. Details about preprocessing are provided in^89^.

#### Pseudo-Continuous Arterial Spin Labeling (pCASL)

FD for pCASL data were quantified with AFNI’s 3dvolreg function. With a total of 680 images across all timepoints, 3.4% of participant data exceeded 2 SD above the mean FD across subjects.

#### Fluid-attenuated inversion recovery (FLAIR) MRI

FLAIR data were segmented into normal and hyperintense regions with the Lesion Growth Algorithm from the Lesion Segmentation Toolbox (LST v3.0)^90^ with a kappa threshold of 0.3. As expected, Fig. 14 shows that the oldest age group follows the steepest increase in total WMH volume.

## Usage Notes

The MIVAC dataset extends the NKI-RS initiative into midlife and older adulthood, offering an openly available resource for examining normative and atypical trajectories of brain aging. The dataset is designed for broad application across neuroscience, psychology, gerontology, and public health, among other fields. Since its inception, the NKI-RS has supported the training of emerging scientists at the undergraduate, master, and doctoral levels, as well as advanced research by established investigators, accelerating discovery and fostering the next generation of basic and clinical research. Below we outline key considerations for effective use.

### Data quality

In keeping with NKI-RS open science principles, all data are released without exclusion on the basis of quality. This allows researchers to apply their own quality-control thresholds and to develop or benchmark artifact-correction methods. Phenotypic data are released without removal of outliers, with the expectation that users apply study-specific cleaning and screening procedures appropriate to their research aims.

### Longitudinal considerations

The MIVAC sub study employed a multi-cohort longitudinal design, with repeated measures up to four years. Due to the COVID-19 pandemic, data collection was interrupted during the third annual follow-up period, resulting in substantial missingness for the 4th timepoint. Variables coding visit intervals (e.g., “day lag”) are provided to allow precise modeling of within-person change and practice effects. Users should account for attrition and missing data when conducting longitudinal analyses.

### Phenotypic protocols

Measures were harmonized across prior NKI-RS sub studies to facilitate lifespan analyses. Some measures (e.g., CASI-A, MoCA) were extended to broader age ranges and include item level data for methodological analyses. Users are advised to consult the full End User Protocol (https://rocklandsample.org/mivac-full-end-user-protocol-2) and detailed item-level information in a downloadable file here: https://rocklandsample.org/for-researchers-rockland-sample-i-2011-2022-documentation.

### Cardiorespiratory fitness and physical health measures

This dataset incorporates gold-standard submaximal VO₂max assessments, providing an opportunity to examine modifiable protective factors in aging. Investigators should review cardiorespiratory assessment quality flags when incorporating this assessment into analyses. For blood-based analyte analyses, we recommend (i) log-transforming long-tailed biomarkers, (ii) explicitly modeling fasting status and relevant medications where available, and (iii) reporting both robust (median/IQR) and parametric (mean/SD) summaries.

### End user support

An inquiry response mechanism to ensure “End Users” appropriately understand and access data is maintained to support data use. End users submit inquiries to rocklandsample.enduser@nki.rfmh.org for support with data access, data quality, or documentation; to provide feedback if a technical error is identified; and to document publications using the data resource. Additionally, an End User Response Panel, composed of NKI-RS program investigators with expertise in phenotyping, clinical characterization, database management, and imaging convenes weekly to support NKI-RS staff responses to End Users. NKI-RS research staff monitor email at least weekly and forward questions to the Panel. Since implementation, the Panel has received and jointly responded to 598 unique inquiry threads as of June 2025 (2017: 83; 2018: 62; 2019: 41; 2020: 60; 2021: 81; 2022: 108; 2023: 58; 2024: 56; 2025: 49).

### Intended use

The dataset is well-suited for investigations of normative and atypical trajectories of brain aging beginning in midlife, as well as the role of modifiable health factors, including cardiorespiratory fitness, BMI, sleep, and social networks, in shaping cognitive and neural outcomes. The depth and heterogeneity of the sample also make it a strong resource for methods development targeting artifact detection, denoising, and harmonization across open imaging datasets.

This dataset is adequately powered to detect small to large effects. For example, at n = 348, a multiple regression analysis with 150 predictors provides ~80% power to detect small f^2^-0.15 and > 99% power to detect large (f^2^ = 0.35) effects. Researchers interested in broader lifespan analyses will find additional leverage in the harmonized protocols maintained across NKI-RS sub studies, which support direct comparisons and pooled analyses spanning the full age range of the cohort.

### Neuroimaging data processing

#### pCASL

*Post-labeling delay*. We chose a 1000 ms post-labeling delay (PLD) for pCASL to optimize effective SNR and harmonize with the broader NKI-Rockland Sample lifespan protocol. Given the exponential decay rate of labeled blood, a 1000 ms PLD retains ~55% (e^−1000/1650^) of the labeled bolus versus ~34% (e^−1800/1650^) at the consensus-recommended 1800ms^91^ at 3 T. While longer PLDs improve theoretical SNR through greater label recovery, they simultaneously extend the time window vulnerable to motion-driven subtraction noise. This is a practical concern in a cohort including children and older adults, both of whom tend to be hyperkinetic. CBF analyses are largely restricted to cortical gray matter, where arterial transit times (ATT) are shortest^91,92^. If ATT variability is a specific concern, post-hoc correction using established kinetic modeling pipelines (e.g., BASIL^93,94^) can be applied. Quantitative CBF values should be interpreted as within-study relative measures rather than absolute normative benchmarks.

A note on hematocrit and cerebral perfusion. Hematocrit (Hct) influences the T1 of blood: T1blood = (0.52 × Hct + 0.38)^-1^90^, and the standard ISMRM assumption of a fixed Hct of 43.5% (corresponding to T1blood = 1650 ms at 3 T^91^; introduces systematic CBF overestimation in individuals with lower hematocrit. Hct decreases in later life (e.g^95^.), and women have characteristically lower hematocrit than men (e.g^96^.). However, these individual differences can be accounted for by modeling Hct in individual T1blood estimates using BASIL or equivalent software, and is strongly recommended when comparing male and female participants (e.g^97^.). Individually measured hematocrit values are provided in the NKI-RS labs data (nki_labs_01.csv), enabling researchers to implement this correction directly in their ASL quantification pipelines.

#### Imaging data processing software recommendations

As raw imaging data are provided without preprocessing or derivative computations, users are directed to the following resources for modality-specific processing guidance. For pCASL data, we recommend ASLprep^98^ and BASIL^93,94^. Resting-state fMRI processing is supported by the C-PAC pipeline^99^, and diffusion MRI data can be processed using the protocols described in^100,101^ and QSIprep^82^. FLAIR images, including white matter hyperintensity segmentation, can be processed using the LST toolbox^90^.

### Limitations

Limitations include incomplete longitudinal follow-up due to pandemic-related shutdown, the need for users to implement quality-control and outlier handling, and sex representation skewed toward females. Researchers should interpret findings within the context of these constraints. Please note, while sex representation is uneven, sample sizes remain sufficient for sex-stratified analyses across broader age bands.

Additionally, while recruitment strategies were designed to approximate a community-representative sample for the local region, the MIVAC cohort reflects the demographic profile of the greater Rockland County area, which differs from national averages in a few respects, including higher levels of education and household income and lower representation of Hispanic/Latino individuals and rural communities. These differences may limit the generalizability of findings to more diverse populations. We encourage research groups in other geographic regions, including rural areas, to collect comparable measures using harmonized protocols, enabling future cross-site analyses that combine demographically diverse samples and strengthen the broader applicability of findings from this resource.

## Data Availability

Information for researchers about the Rockland Sample study and how to obtain data can be found on the study website: https://rocklandsample.org/for-researchers. Instructions for access to a static dataset used to generate this data paper and detailed descriptions specific to the MIVAC study are available here: 10.15387/MIVAC Phenotypic data is available on the NKI hosted LORIS platform: https://data.rocklandsample.rfmh.org Step-by-step instructions on how to download neuroimaging data can be found here: https://rocklandsample.org/accessing-the-neuroimaging-data-releases Instructions on the repository website detail how to access the open AWS S3 bucket. BIDS organized data can also be directly accessed through this S3 path:s3://fcp-indi/data/Projects/RocklandSample/RawDataBIDSLatest.

## Code availability

Python scripts written in-house were used to calculate summary scores for a few variables presented in this paper and are listed in variables_used.csv. Both the csv file and the scripts are available on the MIVAC webpage (10.15387/MIVAC) for download.
